# Metabolic Plasticity of *Candida albicans* in Response to Different Environmental Conditions

**DOI:** 10.3390/jof8070723

**Published:** 2022-07-12

**Authors:** Mariana Gallo, Laura Giovati, Walter Magliani, Thelma A. Pertinhez, Stefania Conti, Elena Ferrari, Alberto Spisni, Tecla Ciociola

**Affiliations:** Department of Medicine and Surgery, University of Parma, 43126 Parma, Italy; mariana.gallo@unipr.it (M.G.); laura.giovati@unipr.it (L.G.); walter.magliani@unipr.it (W.M.); elena.ferrari@unipr.it (E.F.); alberto.spisni@unipr.it (A.S.); tecla.ciociola@unipr.it (T.C.)

**Keywords:** *Candida albicans*, fungal adaptability, morphological transition, metabolomics, NMR spectroscopy, yeast-to-hypha transition

## Abstract

The ubiquitous commensal *Candida albicans*, part of the human microbiota, is an opportunistic pathogen able to cause a wide range of diseases, from cutaneous mycoses to life-threatening infections in immunocompromised patients. *Candida albicans* adapts to different environments and survives long-time starvation. The ability to switch from yeast to hyphal morphology under specific environmental conditions is associated with its virulence. Using hydrogen nuclear magnetic resonance spectroscopy, we profiled the intracellular and extracellular metabolome of *C. albicans* kept in water, yeast extract–peptone–dextrose (YPD), and M199 media, at selected temperatures. Experiments were carried out in hypoxia to mimic a condition present in most colonized niches and fungal infection sites. Comparison of the intracellular metabolites measured in YPD and M199 at 37 °C highlighted differences in specific metabolic pathways: (*i*) alanine, aspartate, glutamate metabolism, (*ii*) arginine and proline metabolism, (*iii*) glycerolipid metabolism, attributable to the diverse composition of the media. Moreover, we hypothesized that the subtle differences in the M199 metabolome, observed at 30 °C and 37 °C, are suggestive of modifications propaedeutic to a subsequent transition from yeast to hyphal form. The analysis of the metabolites’ profiles of *C. albicans* allows envisaging a molecular model to better describe its ability to sense and adapt to environmental conditions.

## 1. Introduction

*Candida albicans* is part of the commensal microbiota and an opportunistic pathogen for humans, causing mucosal and systemic infections in susceptible individuals, including elderly, oncologic, transplanted, and immunosuppressed patients [1]. It has an extraordinary ability to survive in disparate conditions, including oxygen- and nutrient-deprived environments [2,3,4]. Adaptation, in turn, influences its pathogenicity by modulating the expression of key virulence factors [5].

*Candida albicans* is a polymorphic fungus [6,7,8], and the yeast-to-hypha transition is a key step for the expression of its pathogenicity due to hyphae’s ability to penetrate tissues [9,10,11,12,13,14], escape phagocytosis, and facilitate macrophages or neutrophils death [15,16]. The transition from budding yeasts to hyphal growth, or vice versa, is influenced by environmental factors, such as neutral pH, nutrients’ limitation, elevated CO_2_, and temperature, as well as by the presence of serum, proline, or N-acetylglucosamine [7,17,18,19,20]. These conditions trigger a tightly regulated network of signaling pathways and transcription factors that activate the expression of specific genes [7,20,21,22,23,24,25]. However, their downstream effects on primary metabolism have not been fully characterized yet.

Recognizing that metabolic profiling provides a direct “functional readout of the physiological state of an organism” [26], an increasing number of metabolomics-based studies have contributed to further understanding the molecular mechanisms that govern the microorganisms’ life [27,28,29]. Yeasts represent the best eukaryotic model, and comparative analyses of their metabolic profiles under different experimental conditions, in the presence of drugs, or when using genetically manipulated strains have provided precious hints at the molecular level [30,31,32,33,34,35,36,37,38,39,40]. The combination of the pathogenic microorganisms’ metabolome with the infection-associated changes in the host’s metabolism is expected to offer a more complete comprehension of the infective process [41,42,43,44,45,46].

Using a metabolomics approach, Han and collaborators described an overall downregulation of *C. albicans* metabolism during yeast-to-hypha transition [47]. In the hyphal form, carbon and nitrogen metabolic pathways are primarily perturbed, and the ATP level is reduced. Some studies explored the metabolic effects of molecules that suppress (phenylethyl alcohol [48]) or induce (N-acetylglucosamine [49]) yeast-to-hypha transition, while a recent transcriptomic and metabolomic study pointed out that the transition of *C. albicans* to its pathogenic form requires metabolic flexibility [50].

We used hydrogen nuclear magnetic resonance (^1^H-NMR) spectroscopy to profile the intracellular and extracellular metabolome of *C. albicans* cells kept in water, yeast extract–peptone–dextrose (YPD), and M199 at selected temperatures. The experiments were carried out in hypoxia to mimic a condition present in most colonized niches. Our aim was to verify if nutrient availability and incubation temperature may select and reprogram specific metabolic pathways, also anticipating a subsequent transition to the pathogenic hyphal state.

## 2. Materials and Methods

### 2.1. Candida albicans Sample Preparation and Quantification

*Candida albicans* SC5314 was the strain used in this study. The reference sample was prepared following a previously described procedure aimed to ensure that all cells are in a similar growth phase [51]. Briefly, yeast cells were cultivated for 24 h at 37 °C on Sabouraud dextrose agar, and then approximately 2 × 10^5^ cells were suspended in 10 mL of YPD (yeast extract 1%, peptone 2%, dextrose 2%) broth. Yeast extract (product code 70161, batch 0001439171) and peptone (product code 70172, batch 0001437644) were purchased from Merck-Millipore (Merck KGaA, Darmstadt, Germany). After incubation at 25 °C for 16 h with orbital shaking (150 rpm), cells were washed with water, resuspended in 10 mL of fresh YPD broth, and incubated with shaking (150 rpm) for 24 h at 37 °C. After a washing step and microscopic counting, cell suspension was adjusted to obtain approximately 5–6 × 10^8^ cells/mL. In this setting, *C. albicans* cells are predominantly in their yeast form. The quantification by microscopic counting confirmed that germ tubes/hyphal cells were <0.5% (an average of 1000 cells were counted in several fields). This suspension was taken as the reference sample (Ca_0_). The number of viable cells in the reference sample was also established by colony forming units (CFU) counting, after incubation at 30 °C for 48 h of Sabouraud agar plates seeded with 50 µL of the suspension diluted 1:150,000. Viable cells in Ca_0_ were 4.8–6.3 × 10^8^ cells/mL.

### 2.2. Culture Conditions and Metabolite Collection

#### 2.2.1. Culture Conditions

Cells from 1 mL of Ca_0_ were resuspended in 1 mL of the selected media and maintained in hypoxic conditions. Cells were incubated in various culture conditions: (a) for 1 h and 6 h in water (pH 5.5) at 37 °C. This is a starving condition that provides information on cell basal metabolism, avoiding any interference from medium composition; (b) for 6 h in YPD (pH 6.5), a glucose and amino acids rich medium, at 30 °C and 37 °C; (c) for 6 h in M199 (pH 7.0, product code M4530, batch RNBD1893, Sigma-Aldrich, St. Louis, MO, USA), a medium with reduced glucose and amino acids concentration, at 30 °C and 37 °C.

Microscopic quantification of cellular morphology and CFU counting were performed for each sample after incubation.

In the adopted conditions, *C. albicans* remains in the yeast form. In M199 at 37 °C, yeast morphology is maintained due to the employed high cell density, but at lower cell density, the yeast-to-hypha transition may take place. To verify this assumption, after 6 h of incubation under each experimental condition, aliquots of the cell suspensions were diluted 1:100 in the corresponding fresh media and, after incubation of 2 h, microscopic quantification of cellular morphology was carried out.

#### 2.2.2. Metabolite Collection

After 6 h of incubation, the pH of culture supernatants decreased to 4.5 in all the experimental conditions.

**Figure 1:**
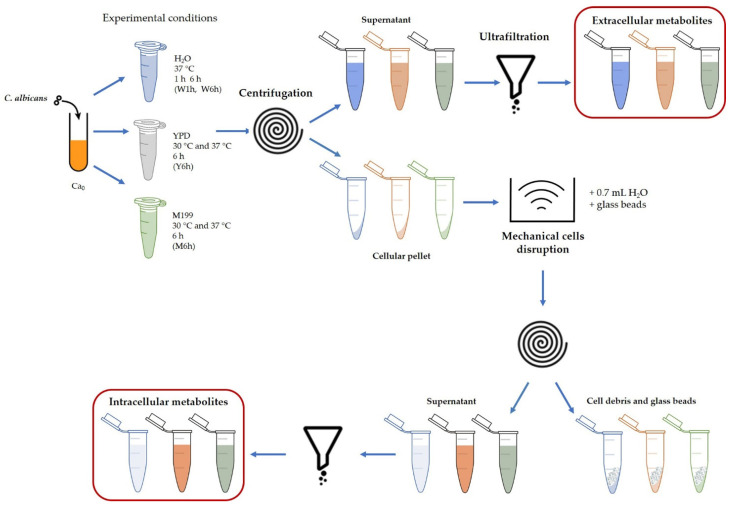
Outline of the sample preparation protocol, designed to collect a set of extracellular and intracellular metabolites (red boxes) for each experimental condition. To obtain a hypoxic environment, incubation was carried out in closed tubes. Intracellular metabolites were also produced from the initial reference culture Ca_0_. W1h and W6h, cells suspension in water at 1 h and 6 h (37 °C); Y6h, YPD culture at 6 h (30 °C and 37 °C); M6h, M199 culture at 6 h (30 °C and 37 °C).

The sample preparation protocol is illustrated in Figure 1.

*Extracellular metabolites:* cells suspended in water or cultured in YPD and M199 media were centrifuged at 12,000× *g* for 5 min at 4 °C. The supernatants were protein depleted by ultrafiltration at 4000× *g* for 30 min at 4 °C (Amicon Ultra-2 Centrifugal Filter Unit, membrane cut-off 10 kDa, Merck KGaA, Darmstadt, Germany). YPD and M199 fresh media were ultrafiltered at 4000× *g* for 30 min at 4 °C to be used as reference.

*Intracellular metabolites:* pelleted cells, suspended in 0.7 mL water, were mechanically disrupted with glass beads in an ultrasonic bath (Branson Ultrasonics, Brookfield, CT, USA). The lysates were centrifuged at 12,000× *g* for 5 min at 4 °C and protein depleted by ultrafiltration at 4000× *g* for 60 min at 4 °C (Amicon Ultra-2 Centrifugal Filter Unit, membrane cut-off 10 kDa). This procedure was used for all the experimental conditions and the reference culture (Ca_0_).

### 2.3. Sample Preparation for ^1^H-NMR Measurements

NMR samples (final volume 600 μL) were prepared using aliquots of ultrafiltered lysates (intracellular metabolites), supernatants (extracellular metabolites), and fresh media (references for extracellular metabolites). They contained 25 mM phosphate buffer pH 7.4, 2.5% D_2_O for signal lock, and 1.45 mM 3-trimethylsilyl propanoic acid (TSP) as chemical shift reference (0.00 ppm) and quantitative internal standard.

### 2.4. ^1^H-NMR Experiments and Analysis

One-dimensional ^1^H-NMR spectra were acquired at 25 °C with a JEOL 600 MHz ECZ600R spectrometer (JEOL USA Inc., Peabody, MA, USA) using the first increment of the pulse sequence NOESY-presat, 128 scans, sweep window 15 ppm, 128 k points, and relaxation delay of 5 s. The spectra were processed and analyzed with the Chenomx NMR suite 7.6 software (Chenomx Inc., Edmonton, AB, Canada), zero-filling to 256 k points, and using a line broadening of 0.5 Hz.

### 2.5. Metabolite Concentrations and Statistical Analysis

The intracellular metabolite concentrations were normalized to the corresponding number of total cells. Each metabolite concentration is the average of three replicates. The relative standard deviation (RSD) was calculated for the three concentrations of each detected metabolite. The average RSD values of all the metabolite concentrations, measured across all the samples, were 21% and 12% for the intracellular and extracellular metabolites, respectively.

Relative changes in intracellular metabolite concentrations, in different experimental conditions, were calculated as the ratio between their concentrations. Alternatively, relative changes were expressed as the ratio between metabolite concentrations and the corresponding values measured in Ca_0_.

Variations in extracellular metabolite concentrations measured in YPD or M199 were expressed as percentage change with respect to the fresh medium composition. Negative values indicate metabolites consumed by the cells, while positive values refer to metabolites secreted by the cells into the medium.

Multivariate statistical analysis (partial least squares—discriminant analysis, PLS-DA) was carried out using the platform MetaboAnalyst 5.0 (https://www.metaboanalyst.ca, accessed on 21 March 2022) [52]. Metabolite intracellular concentration data were further normalized by the median and auto-scaled prior to analysis. MetaboAnalyst suite was also used to perform the pathway analysis, based on the KEGG *Saccharomyces cerevisiae* pathway database. The *x*-axis of the pathway analysis plot corresponds to the pathway impact value computed by pathway topological analysis, and the *y*-axis is the −log of the *p* value (−log10(p)) from pathway enrichment analysis. The most significantly impacted pathways appear in the top right region of the plot.

## 3. Results

### 3.1. Overview of C. albicans Metabolome in Different Culture Conditions

For all selected experimental conditions, due to the high cell density, the number of fungal cells did not vary considerably after 6 h of incubation. Microscopic quantification confirmed a largely predominant yeast morphology (yeast cells > 99%) in all the tested conditions. However, it is important to note that, when *C. albicans* suspensions were diluted 1:100 in fresh medium, the yeast-to-hypha transition took place, as expected, only for M199 cultures incubated at 37 °C.

Figure S1 shows representative ^1^H-NMR spectra obtained from *C. albicans* samples incubated in different conditions. Using ^1^H-NMR spectroscopy, 49, 59, and 62 intracellular metabolites were identified for *C. albicans* incubated in water, YPD, and M199, respectively. A lower number of metabolites was found in the supernatants (extracellular metabolites): 22, 38, and 43, in water, YPD, and M199, respectively. Overall, the identified intracellular and extracellular metabolites were grouped into the following categories: amino acids (n = 20), amino acid derivatives (n = 7), molecules associated with lipid metabolism (n = 11), pyruvate/tricarboxylic acid (TCA) metabolism (n = 6), carbohydrate metabolism (n = 6), purine and pyrimidine metabolisms (n = 8), and others (n = 8).

**Figure 2:**
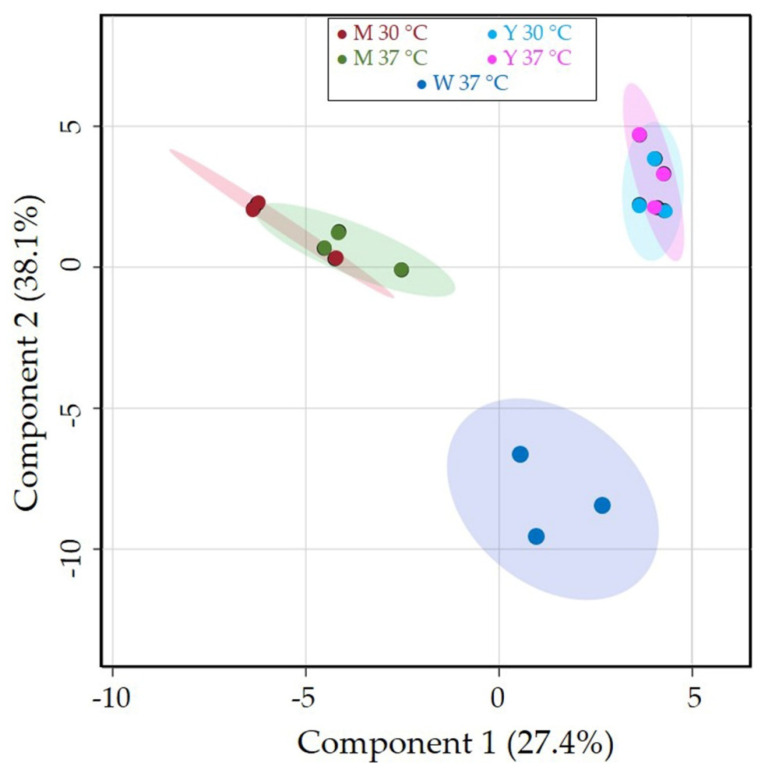
Partial least squares—discriminant analysis score plot of intracellular metabolomes obtained from *C. albicans* at 37 °C in water (W, blue circles) and at 30 °C and 37 °C in YPD (Y, cyan and magenta circles) and M199 (M, red and green circles) media. Colored ellipses represent the 95% confidence regions of each clustering.

PLS-DA was applied on *C. albicans* intracellular metabolite datasets, obtained after incubation at 37 °C in water, and at 30 °C and 37 °C in YPD and M199 media. PLS-DA scores plot resulted in three well-separated clusters (Figure 2) that reflect distinct metabolome compositions depending on nutrient availability and highlight, for both YPD and M199, a close similarity at the different incubation temperatures.

### 3.2. The Metabolome of C. albicans in Water, YPD, and M199

#### 3.2.1. Intracellular Metabolites of *C. albicans* Incubated in Water at 37 °C

**Figure 3:**
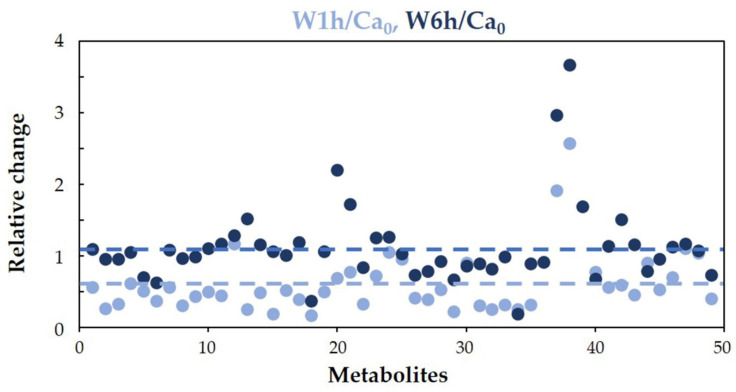
Distribution of the relative changes in intracellular metabolite concentrations, after incubation in water at 37 °C for 1 h (sky blue) or 6 h (dark blue), compared to the reference condition (Ca_0_). Intracellular concentrations of metabolites were normalized per total cell number. Broken lines indicate the mean value of the ratios W1h/Ca_0_ and W6h/Ca_0_ calculated for all the identified metabolites (0.6 for 1 h and 1.1 for 6 h incubation). Each dot represents a quantified metabolite; dots aligned vertically correspond to the same metabolite (1 h and 6 h).

In water, an overall downregulation of the cellular metabolism was observed after 1 h incubation, with an average ratio W1h/Ca_0_ of about 0.6. However, after 6 h, there was a recovery to the initial conditions, with an average ratio W6h/Ca_0_ near to 1 (Figure 3). Nonetheless, the concentration of some intracellular metabolites remained significantly altered with respect to the initial condition (Figure S2). Glutamine, β-alanine, arabinitol, UDP-glucuronate, and glycerol decreased, while ornithine, proline, reduced glutathione, hypoxanthine, uracil, uridine, and O-phosphocholine increased.

The drop in glutamine and β-alanine suggests their use as nitrogen and carbon sources for anabolic processes; the increase in ornithine levels can be correlated with the consumption of glutamate, a possible additional nitrogen source. The buildup of reduced glutathione reflects the limited oxygen availability. As for the increase in uracil and uridine, it indicates an alteration of pyrimidine metabolism that might have a connection with the decrease in UDP-glucuronate. Hypoxanthine increase can be associated with ATP depletion, while the decrease in glycerol may be interpreted as the response of the cells to counter the osmotic stress.

#### 3.2.2. Intracellular Metabolites of *C. albicans* Incubated in YPD and M199

Having observed that *C. albicans* cells, in water, require about 6 h to recover the initial metabolic content, we adopted that incubation time for assaying YPD and M199 cultures.

**Figure 4:**
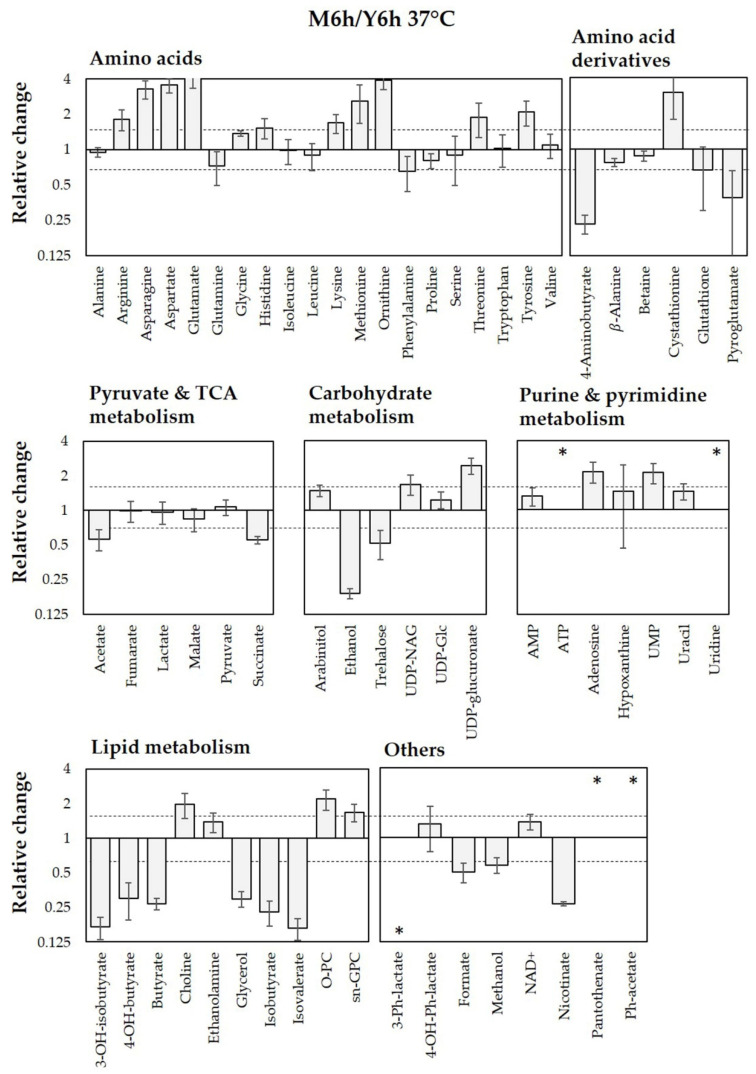
Relative change in intracellular metabolite concentrations after 6 h of incubation at 37 °C in M199 (M6h) compared to YPD (Y6h). Concentrations were normalized per total cell number and metabolite median concentration. Relative changes are expressed as metabolites’ concentration ratio (M6h/Y6h). Ratios higher than 1.5 or lower than 0.66 (indicated by dashed lines) were considered representative of significant changes. TCA: tricarboxylic acid; O-PC: O-phosphocholine; sn-GPC: *sn*-glycerophosphocholine; UDP-NAG: uridine diphosphate-N-acetylglucosamine; UDP-Glc: uridine diphosphate-glucose; UMP: uridine monophosphate; Ph: phenyl; OH: hydroxy. Y axis is in log_2_ scale. The asterisk (*) indicates that the metabolite is present only in one of the two culture conditions (M199 if in the upper part, YPD if in the lower part of the graph).

By comparing the intracellular metabolite concentrations obtained at 37 °C in the two media, we found that the most relevant differences were related to the amino acid pool, nicotinate, and some metabolites involved in lipid and purine/pyrimidine metabolisms (Figure 4).

**Figure 5:**
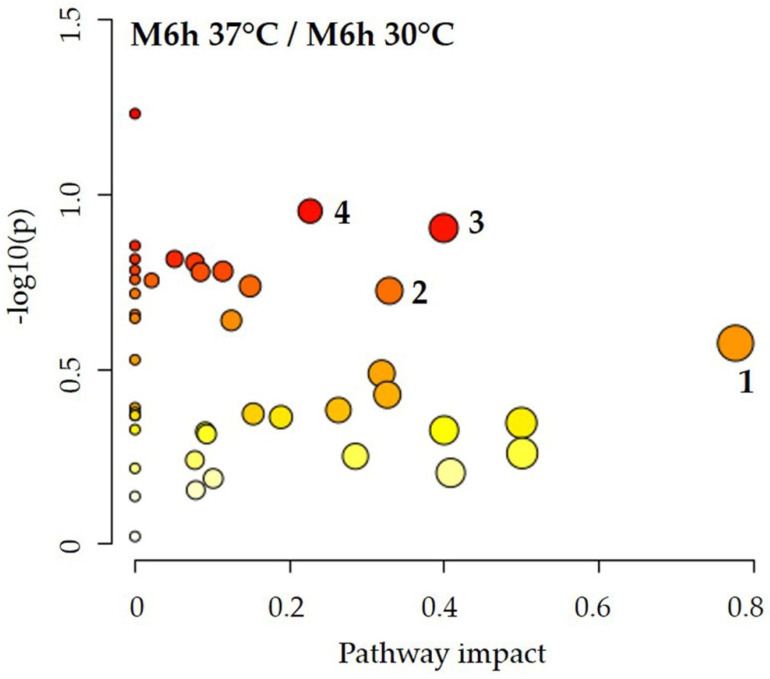
Pathway analysis showing the enriched pathways that arose from comparing the intracellular metabolomic datasets obtained in M199 at 30 °C and 37 °C. Matched pathways are displayed as circles. The color of each circle is based on *p* values (a darker color indicates more significant differences in metabolite concentrations of a specific pathway), whereas the size of the circle corresponds to the pathway impact score. The most impacted pathways, despite the low significance (*p* > 0.05), are: (1) alanine, aspartate, and glutamate metabolism; (2) arginine and proline metabolism; (3) nicotinate and nicotinamide metabolism; (4) pyrimidine metabolism. *p* values denote enrichment of the entire pathway by Fisher’s exact test.

The transition to the hyphal state in *C. albicans* cells grown in M199 takes place at 37 °C (but not at 30 °C) at a cell density lower than that adopted in this study. Based on this observation, we performed the pathway analysis by comparing the intracellular metabolic content obtained in M199 at 30 °C and 37 °C (Figure 5). Though with low statistical significance (*p* > 0.05), Figure 5 highlights alteration for some pathways: (1) alanine, aspartate, and glutamate metabolism; (2) arginine and proline metabolism; (3) nicotinate and nicotinamide metabolism; (4) pyrimidine metabolism. In addition, Figure S3 shows that, although marginally, the concentration of a good number of metabolites changes, including some metabolites related to lipid and energy metabolism.

#### 3.2.3. Extracellular Metabolites of *C. albicans* in Water, YPD, and M199 at 37 °C

In water, the most abundant secreted metabolites, with concentrations higher than 50 µM, were acetate, lactate, arabinitol, ethanol, glycerol, and formate (Table S1). Their concentrations nearly doubled from 1 h to 6 h incubation. It is worth noting that the data are consistent with the recognized *C. albicans* sensitivity to osmotic stress and support the role of glycerol as a molecule of choice to counteract this event [53].

**Figure 6:**
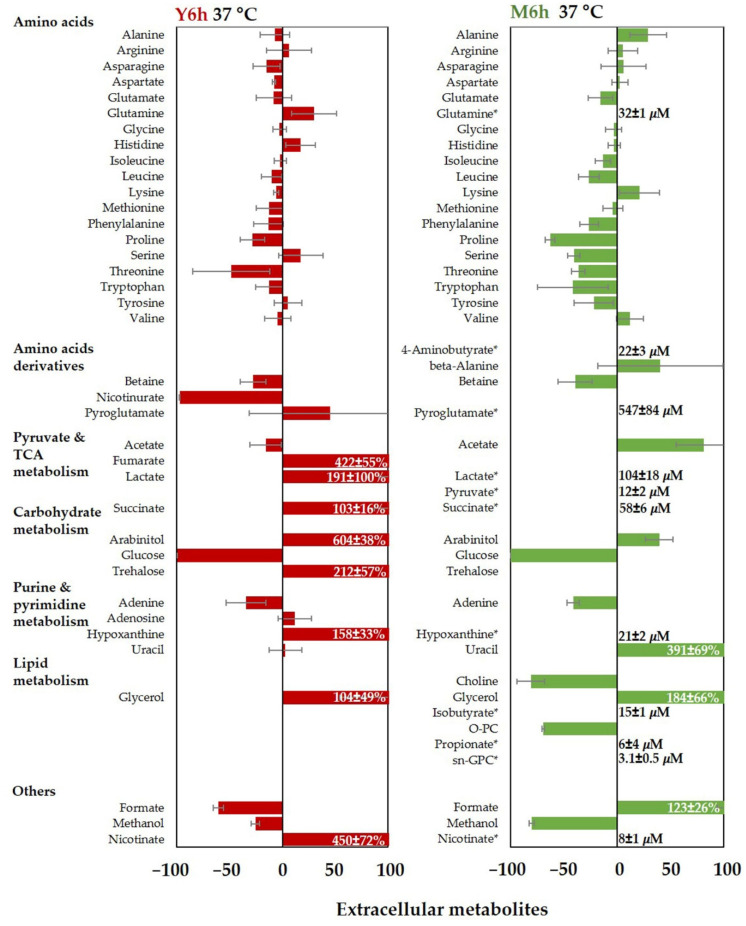
Changes in extracellular metabolite concentrations after 6 h of incubation at 37 °C in YPD (Y6h, red) and M199 (M6h, green). Data are reported as percentage variation of the metabolite concentrations with respect to fresh medium composition. Negative values correspond to metabolites extracted from the medium, while positive values correspond to metabolites released into the medium. TCA: tricarboxylic acid; O-PC: O-phosphocholine; sn-GPC: *sn*-glycerophosphocholine. The asterisk (*) indicates that the metabolite is present in the supernatant but is absent in the fresh medium, and its measured concentration is reported.

Figure 6 shows that in YPD, the net amino acid influx in yeast cells is low while glucose is totally transported into the cells, suggesting a limited use of amino acids when a high amount of glucose is provided (78.6 mM). In addition to glucose and amino acids, *C. albicans* cells use amino acid derivatives, adenine, formate, acetate, and methanol. Glutamine, histidine, and serine, instead, are the main secreted amino acids. Other metabolites secreted in the supernatants are fumarate, lactate, succinate, arabinitol, glycerol, trehalose, hypoxanthine, and nicotinate.

On the contrary, when incubated in M199, *C. albicans* makes extensive use of amino acids, possibly because of the reduced glucose availability (4.7 mM). Figure 6 shows a significant influx of glutamate, isoleucine, leucine, phenylalanine, proline, serine, threonine, tryptophan, and tyrosine, while alanine, lysine, and valine are secreted. Among the secreted metabolites, we find pyroglutamate, acetate, succinate, glycerol, and formate. Lactate is also present in the supernatant, although at lower concentrations as compared to YPD, suggesting a reduced fermentative activity.

These data are consistent with the corresponding intracellular metabolome and suggest that, while *C. albicans* is metabolically active in both media, the differential use of free amino acids is associated with glucose availability.

### 3.3. Candida albicans Energy Metabolism

*Candida albicans* obtains energy primarily from glucose. The data reported in Table 1 indicate that, both at 30 °C and 37 °C, the available glucose is completely metabolized to ethanol after 6 h in YPD and M199, a result consistent with data previously obtained in hypoxic conditions [20]. Interestingly, in both media, the ethanol production exceeds the amount obtainable from the available glucose, confirming that other molecules, such as amino acids, are being used.

In water, in the absence of exogenous nutrients, the production of ethanol is limited, and we envisage it is produced from intracellular sources. These results clearly point to the ability of *C. albicans* to reprogram, in different ways, its energetic metabolism as a function of nutrient availability.

## 4. Discussion

The complex commensal lifestyle of *C. albicans* depends on its ability to survive in different niches, such as oral cavity, gut, and genital tract, where yeast cell fitness is governed by nutrient accessibility, local microbiota, and host immune defenses [54]. Accepting the notion that hypoxic conditions are quite common in most of the colonized niches, another important aspect of *C. albicans* lifestyle is its capacity to adapt to environments with diverse oxygen availability.

These factors influence not only *C. albicans* fitness but also its possible transition from the commensal yeast form to the pathogenic hyphal state. The close interconnection of those factors hampers the possibility to pin down the role of each of them in affecting both gene transcription and metabolism.

Our goal was to investigate how nutrient availability and growth temperature affect specific metabolic pathways and if the metabolic adaptation to particular environmental conditions might set the stage for a subsequent transition to the pathogenic hyphal state. To minimize the effect of other factors, we set up experimental conditions where cell density allows *C. albicans* to be metabolically active but not proliferating. In addition, we limited the oxygen availability to mimic a condition present in most infection sites.

*Candida albicans*, as many other yeasts, in the absence of nutrients, as in water, relies on autophagy and recycles endogenous macromolecules to maintain oxidative activity and viability for several weeks before an inevitable decline [55]. In our experimental conditions, the metabolite content decreased after 1 h incubation at 37 °C, recovering the initial levels after 6 h (Figure 3).

A study, using *C. glabrata* suspended in water at high density, showed that cells release substrates to counter the hypo-osmotic stress [56]. Moreover, it is recognized that *C. albicans* upregulates the production of glycerol and arabinitol [57,58]. Consistently, we found that, already after 1 h starvation, the intracellular glycerol and arabinitol levels decreased as compared to the Ca_0_ sample (W1h/Ca_0_, Figure S2), increasing in the supernatant. After 6 h, however, we observed a recovery of the intracellular concentration (Figure 3 and Figure S2) with a net increase in the glycerol concentration in the supernatant (Table S1). Overall, the data support the role of polyols in the adaptation to hypo-osmotic stress [57,58].

A previous study on the metabolic changes induced by hypoxic conditions in *C. albicans* cultured in YPD highlighted a stimulation of the glycolytic cycle and a repression of the TCA cycle, with a reduction in the ATP level and a concomitant production of ethanol [20]. Our data (Table 1) show that after 6 h in both media, at 30 °C and 37 °C, glucose is fully metabolized. Interestingly, they also clearly highlight that there is a general overproduction of ethanol. In fact, *C. albicans* initially activates the glycolytic route as the main pathway to generate energy and the intermediates necessary for anabolic processes, but, once the glucose supply is finished, it possibly switches from glycolysis to gluconeogenesis and glyoxylate cycle [59]. This ability is particularly relevant for *C. albicans* cultured in M199, where glucose concentration is quite low.

**Figure 7:**
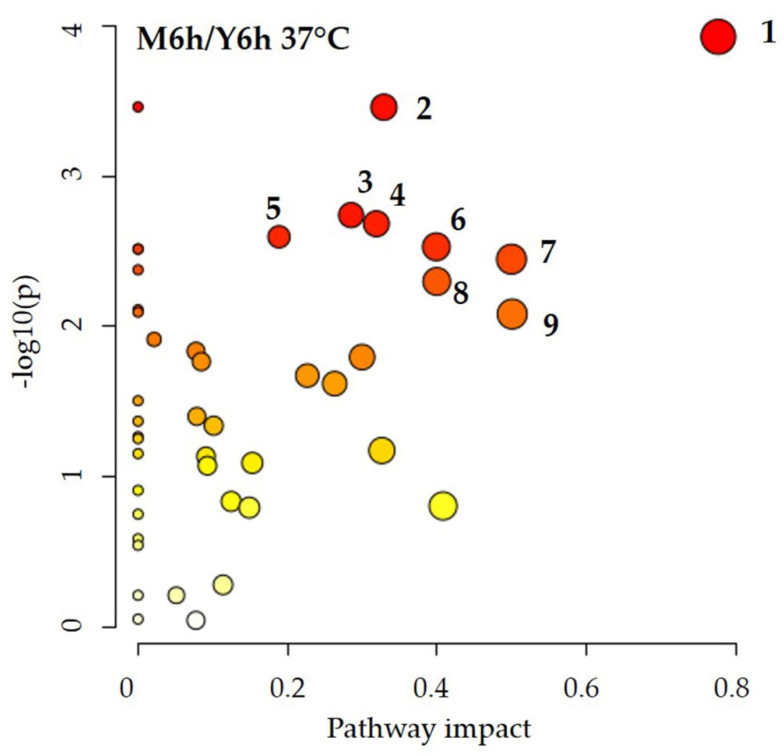
Pathway analysis showing the significantly enriched pathways that arose from comparing the metabolomic datasets obtained in M199 and YPD at 37 °C. Matched pathways are displayed as circles. The color of each circle is based on *p* values (a darker color indicates more significant differences in metabolite concentrations of a specific pathway), whereas the size of the circle corresponds to the pathway impact score. The most significantly impacted pathways (*p* < 0.01), with high statistical significance scores, are: (1) alanine, aspartate, and glutamate metabolism; (2) arginine and proline metabolism; (3) glycerolipid metabolism; (4) arginine biosynthesis; (5) glyoxylate and dicarboxylate metabolism; (6) nicotinate and nicotinamide metabolism; (7) β-alanine metabolism; (8) butanoate metabolism; (9) glutathione metabolism. *p* values denote enrichment of the entire pathway by Fisher’s exact test.

Having verified that, in our experimental conditions, *C. albicans* in M199 does not exhibit significant metabolic differences when incubated at 30 °C or 37 °C (Figure 5 and Figure S3). To investigate the effect of media composition, we performed the pathway analysis using the intracellular metabolite concentrations obtained from *C. albicans* cultured at 37 °C in YPD and M199 (Figure 7).

The metabolic pathways most significantly altered are: (1) alanine, aspartate, and glutamate metabolism; (2) arginine and proline metabolism; (3) glycerolipid metabolism. The results are consistent with the relative changes in intracellular metabolite concentration (Figure 4). With respect to the amino acid metabolism, proline level is reduced in M199 as compared to YPD (Figure 4), suggesting it enters the metabolic pathways responsible for nitrogen assimilation and/or its conversion to glutamate [22], which in fact increased together with aspartate and asparagine. This result is in line with data reporting that proline catabolism is required to sustain the energy demand for hyphal growth [60]. The concomitant decrease in GABA can be related as well to glutamate production (Figure 4). With respect to glycerolipid metabolism, an increase in choline, ethanolamine, and O-phosphocholine level may be linked to a phospholipid rearrangement: a process compatible with morphological and functional cell transitions [8,20]. Finally, ATP accumulation (Figure 4) is acknowledged to stimulate hyphal morphogenesis via the protein kinase A pathway [60].

It is known that *C. albicans,* grown in M199 at 37 °C, undergoes a yeast-to-hypha transition, while this event does not happen for cells cultured in YPD at the same temperature or in M199 at 30 °C. Our data, obtained in M199 and in a condition where that transition is hampered, do not reveal significant metabolism alterations when comparing the intracellular metabolome at 30 °C and 37 °C (Figure 5). However, we hypothesize that the differences in the corresponding metabolite concentrations (Figure S3) might represent a hint of the metabolic modifications propaedeutic to a subsequent transition to the hyphal form.

## 5. Conclusions

To better understand how *C. albicans* can adapt to environmental changes, we examined the intracellular and extracellular metabolomes in different incubation conditions under hypoxia.

In water, the main objective of the cells is to remain viable. Therefore, they activate all internal mechanisms necessary, primarily, to cope with energy needs and to counter environmental stress, e.g., osmotic stress.

YPD and M199 provide glucose, yeast’s preferred energy source. When glucose ends, *C. albicans* expresses its metabolic plasticity by switching to alternative metabolic pathways, the most affected being: (i) alanine, aspartate, glutamate metabolism, (ii) arginine and proline metabolism, and (iii) glycerolipid metabolism.

We believe that the present analysis improves the description of *C. albicans* ability to sense and adapt to diverse environmental conditions.

## Figures and Tables

**Table 1:** Glucose availability and ethanol net production in water, YPD, and M199.

Medium	Glucose	Ethanol (mM) 6 h—30 °C			Ethanol (mM) 6 h—37 °C		
	(mM)	Extracellular	Intracellular ^#^	Production ^§^	Extracellular	Intracellular ^#^	Production ^§^
water	-				3.6	1.1	
YPD	78.6	159.6	6.2	105.5%	168.0	6.5	111.0%
M199	4.7	8.2	2.1	109.6%	9.7	2.0	124.5%

^#^ The intracellular concentration of ethanol is calculated net of its concentration in Ca_0_ sample. ^§^ Ethanol production is expressed as the percentage of the maximum production obtainable from the glucose contained in fresh medium.

## Data Availability

The data presented in this study are available in the article and Supplementary Materials.

## Supplementary Materials

The following are available online at https://www.mdpi.com/article/10.3390/jof8070723/s1, Figure S1: Illustrative examples of the ^1^H-NMR spectra of *Candida albicans* samples (extracellular and intracellular metabolites); Figure S2: Relative changes in intracellular metabolite concentrations after incubation in water at 37 °C for 1 h and 6 h, compared to the reference condition (Ca_0_); Figure S3: Relative changes in intracellular metabolite levels measured after 6 h of incubation in M199 at 37 °C as compared to 30 °C; Table S1: Extracellular metabolites identified in water at 37 °C after 1 and 6 h of incubation.
