# Supplementary material for: Metabolic Plasticity of Candida albicans in Response to Different Environmental Conditions
Source: J Fungi (Basel). 2022 Jul 12;8(7):723. doi: 10.3390/jof8070723 (PMC9322845; doi:10.3390/jof8070723)
Supplement: Supplementary file 1 [file jof-08-00723-s001.zip › jof-1712272-supplementary.pdf]

Metabolic plasticity of *Candida albicans* in response to different environmental conditions

Mariana Gallo, Laura Giovati, Walter Magliani, Thelma A. Pertinhez *, Stefania Conti *, Elena Ferrari, Alberto Spisni and Tecla Ciociola

Department of Medicine and Surgery, University of Parma, 43126, Parma, Italy
* Correspondence: T.A. P., thelma.pertinhez@unipr.it; +390521033825
S. C., stefania.conti@unipr.it; +390521033492

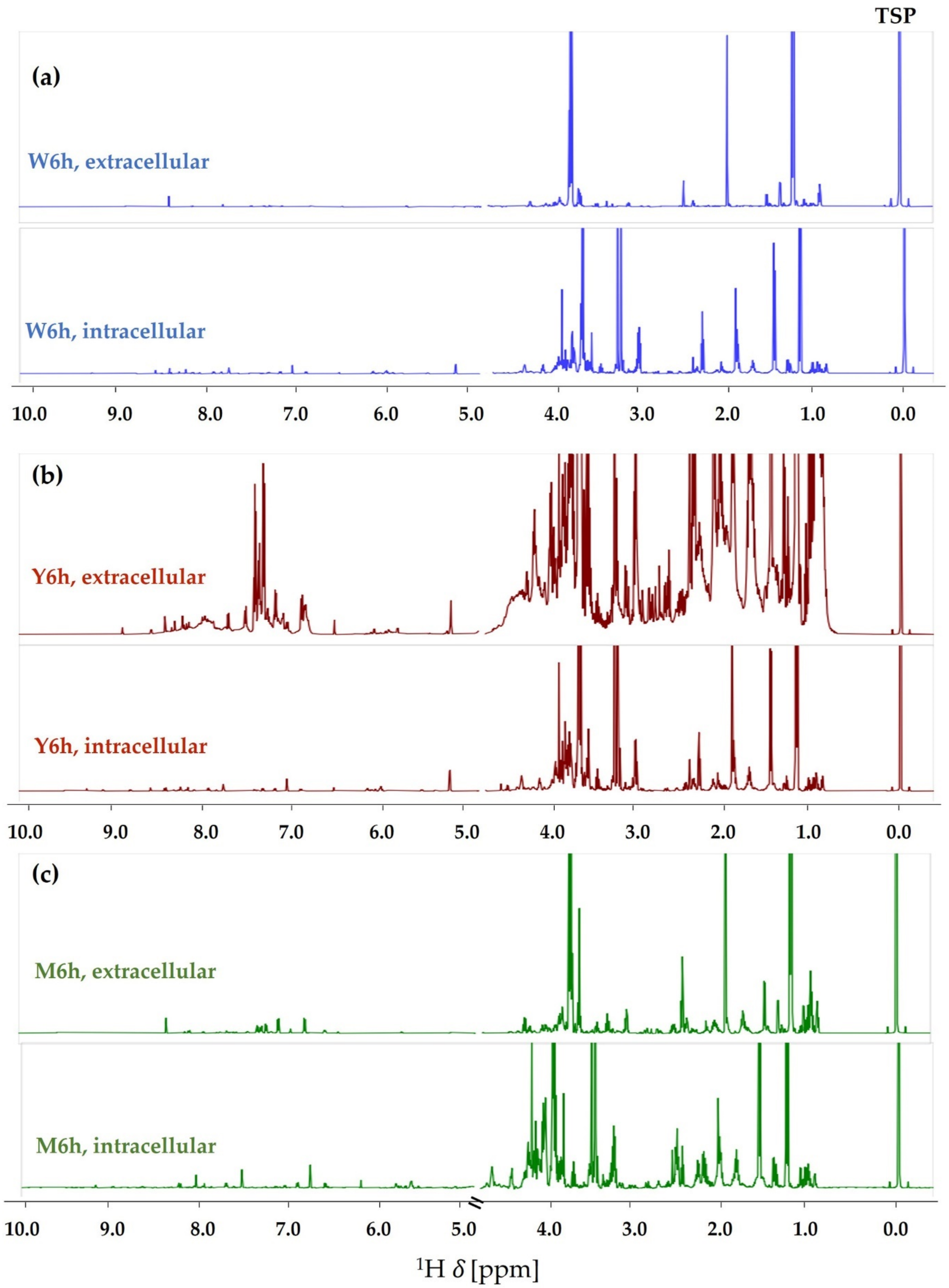

Figure S1. Illustrative examples of the ^{1}H-NMR spectra of *Candida albicans* samples (extracellular and intracellular metabolites). Incubation for 6 h in (**a**) water at 37 °C (W6h); (**b**) YPD at 30 °C (Y6h); (**c**) M199 at 37 °C (M6h). TSP, 3-trimethylsilyl propanoic acid.

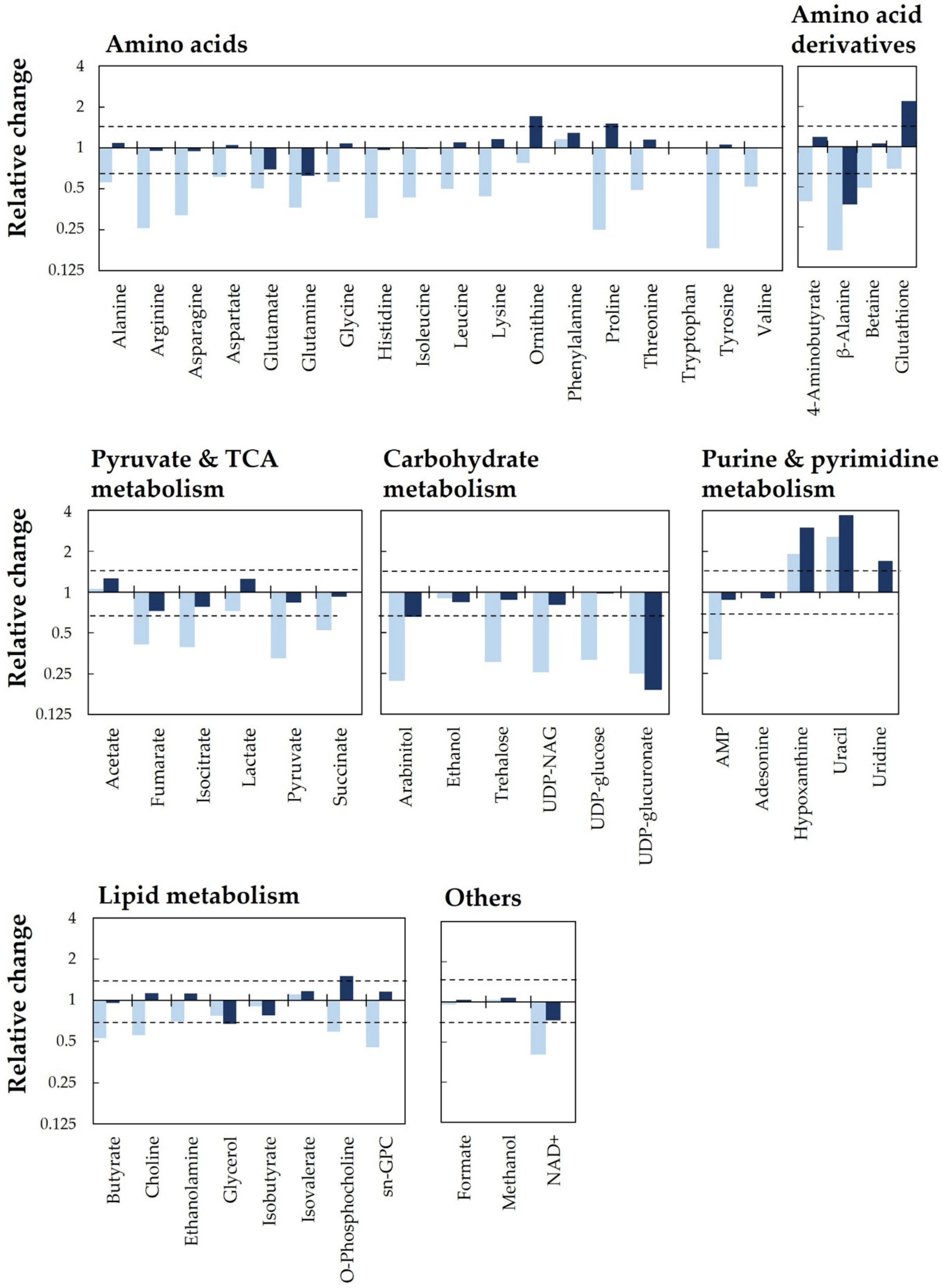

Figure S2. Relative changes in intracellular metabolite concentrations after incubation in water at 37 °C for 1 h (W1h, sky blue) and 6 h (W6h, dark blue) compared to the reference condition (Ca_0). Intracellular concentrations of metabolites were normalized per total cell number. Ratios higher than 1.5 or lower than 0.66 values (indicated by dashed lines) were considered representative of significant changes. TCA: tricarboxylic acid; sn-GPC: *sn*-glycerophosphocholine; UDP-NAG: Uridine diphosphate-N-acetylglucosamine. Y axis is in $\log_2$ scale.

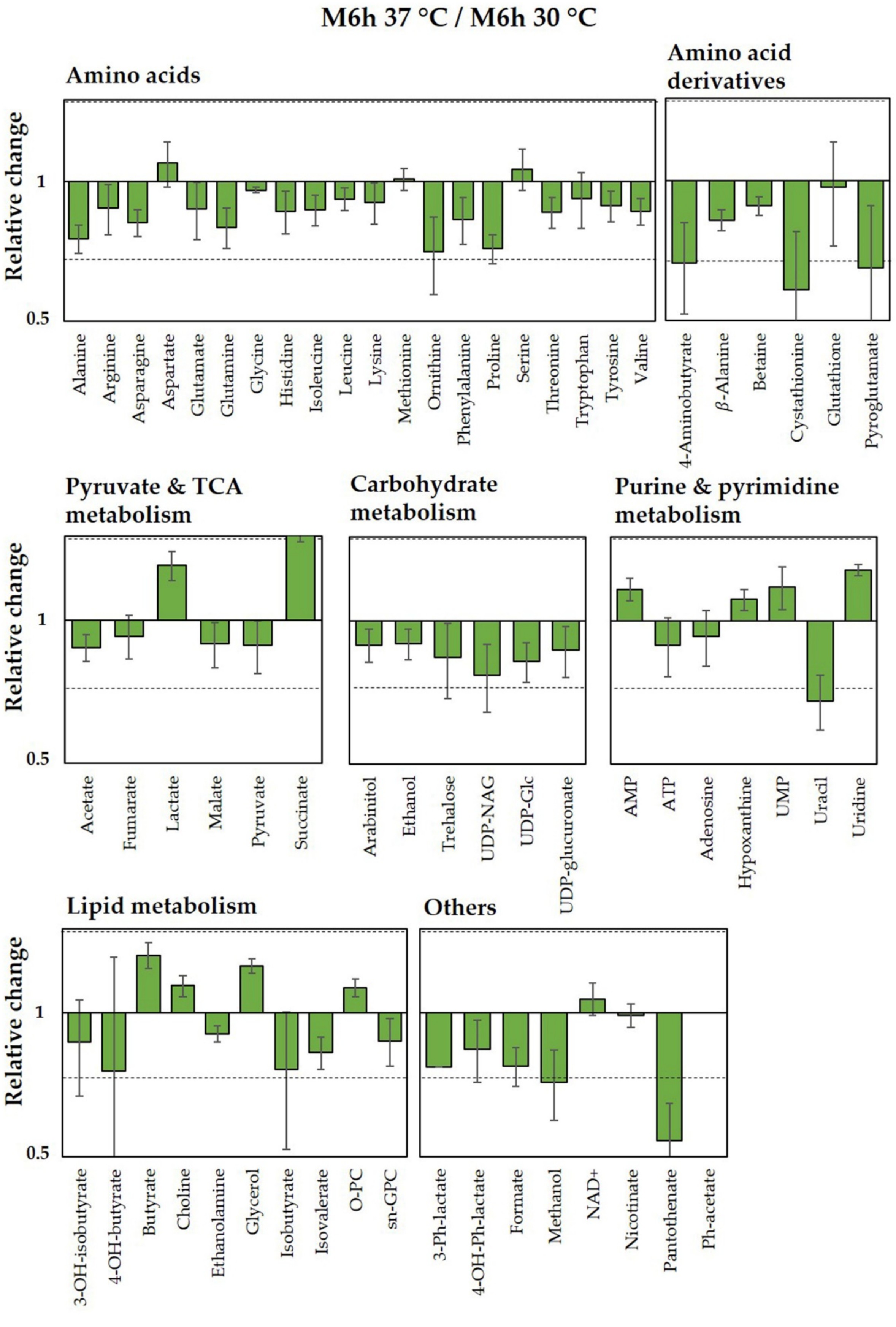

Figure S3. Relative changes in intracellular metabolite levels measured after 6 h of incubation in M199 (M6h) at 37 °C as compared to 30 °C. Concentrations were normalized per total cell number and the median concentration. Ratios higher than 1.5 or lower than 0.66 values (indicated by dashed lines) were considered representative of significant changes. TCA: tricarboxylic acid; UDP-NAG: Uridine diphosphate-N-acetylglucosamine; UDP-Glc: Uridine diphosphate-glucose; O-PC: O-phosphocholine; sn-GPC: *sn*-glycerophosphocholine; OH: hydroxy; Ph: phenyl. Y axis is in log_2 scale.

Table S1. Extracellular metabolites identified in water at 37 °C after 1 and 6 h of incubation.

Metabolites	Concentration [μM] 1 h	6 h
**Amino acids**		
Alanine	15.4	61.5
Glutamate	7.0	12.2
Glutamine	7.8	14.3
Isoleucine	1.5	6.5
Leucine	2.9	10.9
Phenylalanine	5.0	11.4
Threonine	3.9	9.0
Tyrosine	2.5	10.6
Valine	4.0	15.8
**Amino acid derivatives**		
Betaine	1.7	5.5
**Pyruvate & TCA metabolism**		
Acetate	213.8	476.4
Fumarate	1.9	3.0
Lactate	25.7	98.9
Succinate	20.2	42.8
**Carbohydrate metabolism**		
Arabinitol	52.5	73.4
Ethanol	2214.0	3582.0
**Pyrimidine metabolism**		
Uracil	-	17.3
**Lipid metabolism**		
Isobutyrate	11.5	16.3
Glycerol	78.8	162.2
**Others**		
Formate	53.2	115.6
Methanol	3.3	9.8
Nicotinate	-	6.2
